# Protective effect of phytogenic plus short and medium-chain fatty acids-based additives in enterotoxigenic *Escherichia coli* challenged piglets

**DOI:** 10.1007/s11259-022-09945-0

**Published:** 2022-05-26

**Authors:** Valentina Caprarulo, Lauretta Turin, Monika Hejna, Serena Reggi, Matteo Dell’Anno, Pietro Riccaboni, Paolo Trevisi, Diana Luise, Antonella Baldi, Luciana Rossi

**Affiliations:** 1 Department of Health, Animal Science and Food Safety, University of Milan, 26900 Lodi, Italy; 2 Department of Veterinary Medicine, University of Milan, 26900 Lodi, Italy; 3 Department of Agricultural and Food Sciences, University of Bologna, 40126 Bologna, Italy

**Keywords:** Pig, Phytochemicals, Feed additives, Alternatives to antibiotics, Fatty acids, *Escherichia coli*

## Abstract

Post Weaning Diarrhea (PWD) is the most important multifactorial gastroenteric disease of the weaning in pig livestock. Phytogenic (PHY) natural extracts are largely studied as alternatives to antibiotic treatments in combating the global concern of the antimicrobial resistance. The aim of this study was to evaluate the protective effect of innovative phytogenic premix with or without short and medium chain fatty acids (SCFA and MCFA) in O138 *Escherichia coli* challenged piglets. Twenty-seven weaned piglets were allotted into four groups fed different diets according to the following dietary treatments: CTRL (n = 13) group fed basal diet, PHY1 (n = 7) fed the basal diet supplemented with 0.2% of phytogenic premix, PHY2 (n = 7) fed the basal diet supplemented with 0.2% of phytogenic premix added with 2000 ppm of SCFA and MCFA. After 6 days of experimental diet feeding, animals were challenged (day 0) with 2 × 10^9^ CFU of *E. coli* and CTRL group was divided at day 0 into positive (challenged CTRL + ; n = 6) and negative control group (unchallenged CTRL-; n = 7). Body weights were recorded at -14, -6, 0, 4 and 7 days and the feed intake was recorded daily. *E. coli* shedding was monitored for 4 days post-challenge by plate counting. Fecal consistency was registered daily by a four-point scale (0–3; diarrhea > 1) during the post-challenge period. Tissue samples were obtained for gene expression and histological evaluations at day 7 from four animals per group. Lower average feed intake was observed in CTRL + compared to PHY2 and CTRL during the post-challenge period. Infected groups showed higher *E. coli* shedding compared to CTRL- during the 4 days post-challenge (*p* < 0.01). PHY2 showed lower frequency of diarrhea compared to PHY1 and CTRL + from 5 to 7 days post-challenge. No significant alterations among groups were observed in histopathological evaluation. Duodenum expression of occludin tended to be lower in challenged groups compared to CTRL- at 7 days post-challenge (*p* = 0.066). In conclusion, dietary supplementation of PHY plus SCFA and MCFA revealed encouraging results for diarrhea prevention and growth performance in weaned piglets.

**Supplementary Information:**

The online version contains supplementary material available at 10.1007/s11259-022-09945-0.

## Introduction

Post weaning diarrhea (PWD) is a gastrointestinal multifactorial disease that generally occurs during the first two weeks after the weaning phase. It is one of the most economically-relevant diseases in swine husbandry due to the costs of treatments, reduced growth, and increased of mortality (Bonetti et al. 2021). Although many factors are involved in the development of this disease, PWD is often exacerbated by many enterotoxigenic *Escherichia coli* pathotypes characterized by the presence of virulence factors such as toxins and adhesive fimbriae (Sun and Kim 2017). Bacterial resistance to a wide range of commonly used antibiotics is a global concern and a recent increase in prevalence and severity of PWD required alternative measures for their control (Renzhammer et al. 2020; Dell’Anno et al. 2021a, b). Reducing and replacing antimicrobials in animal farming is a crucial aim of the European policies, even if the mechanisms of cross-species transmission of resistant bacteria and their genetic elements spread from livestock to humans has not been fully understood (Rossi et al. 2014a; Cormican et al. 2017; Tang et al. 2017).

The aim of nutrition is no longer simply to satisfy the nutritional requirements, but also play a key role in the health and welfare of humans and animals (Domínguez Díaz et al. 2020; Grossi et al. 2021). Functional feed additives, which sustain the health status and reduce the risk of pathologies, have thus become fundamental in replacing or reducing antimicrobials in food-producing animals. The dietary inclusion of phytogenics (PHYs), represented by plant secondary metabolites, are largely studied as alternative growth promoters because of their biological properties which include antimicrobial, antioxidant, and nutrigenomic effects on the development of animal (Durmic and Blache 2012; Yang et al. 2015; Lillehoj et al. 2018; Reyes-Camacho et al. 2020). In particular Yan and Kim (2012) observed a significant reduction in fecal *E. coli* count after 1 g/kg of eugenol supplementation in pigs. A blend of oregano, anise, and citrus peel (40 mg/kg diet) supplementation to piglets’ diet has been demonstrated to evolve anti-inflammatory effect by reducing the gene expression of NF-kB and TNFα (Upadhaya et al. 2016). The dietary supplementation of thymol, cinnamaldehyde and menthol have been reported to positively affect the feed digestibility in swine (Maenner et al. 2011; Li et al. 2012).The in vivo effects, resulting from the various biological activities of the PHYs, depend on their structure, dosage, and pharmaco-kinetics, as well as the animal species, productive phase and administration period. For this reason, several combinations of natural extracts are currently studied in order to promote their possible synergistic or complementary effect on animal health. Although PHYs show antimicrobial activity in the gastrointestinal tract against specific pathogens such as *Escherichia coli, Clostridium perfringens* and *Salmonella* spp. (Thacker 2013; Mohammadi Gheisar and Kim 2018), their effectiveness can vary due to the presence and the location of functional hydroxyl and phenolic terpenoids (Dubreuil 2013). Rational combinations of PHYs have been studied in order to increase the spectrum of beneficial activities. In addition, the synergistic or complementary effect of PHYs with other compounds leads to various beneficial activities of several compounds, especially organic acid (OA). Amongst feed additives with antimicrobial activities, organic acids, in particular short-chain fatty acids (SCFAs) and medium-chain fatty acids (MCFAs), have a strong antimicrobial activity and are key to modulating intestinal health and improving animal performance (Ferronato and Prandini 2020; Jackman et al. 2020). SCFAs and MCFAs regulate the growth and virulence of enteric pathogens, such as enterohemorrhagic *E. coli*, *Klebsiella* and *Salmonella* (Zhang et al. 2020). They damage the bacterial structure and in some cases separate the inner and outer membranes (Hanczakowska 2017) and thus increase the concentration of IgG and IgM in piglets challenged with enterotoxigenic *Escherichia coli* (ETEC) strains (Han et al. 2020). A synergistic antimicrobial effect has been observed in the combination of PHYs and organic acids in vitro (Costa et al. 2013). However, the effect of their dietary supplementation on pigs' growth and the optimization of the inclusion level for diarrhea prevention against major pathogens of weaned piglets has not been fully investigated. Therefore, it was hypothesized that the dietary supplementation of phytogenic additive with or without organic acids could prevent or limit the detrimental effects of enterotoxigenic *Escherichia coli* infection improving animal health status.

The aim of this study was thus to evaluate the protective effect against O138 *E. coli* F18 + infection of an innovative phytogenic premix composed by caraway oil, lemon oil, clove, cinnamon, nutmeg, onion, pimento, orange peel, peppermint and chamomile powder with and without short and medium chain fatty acids in weaned piglets’ diet.

## Materials and methods

### Animal selection criteria

The trial was performed at the Experimental Animal Research and Application Centre of University of Milan and was authorized by the Italian Health Ministry (authorization n° 711/-PR) in accordance with EU regulations (Directive 2010/63/EU 2010).

Animals enrolled in the experimental trial were selected from a conventional herd free from contagious diseases (Ex A-list International Office of Epizootic, porcine reproductive and respiratory syndrome, atrophic rhinitis, Aujeszky’s disease, transmissible gastroenteritis, salmonellosis) and without a history of PWD or oedema disease. Sows were assessed for genetic susceptibility to *Escherichia coli* carrying F18 adhesive fimbriae (F18 *E. coli*) by screening the fucosyltransferase 1 (FUT1) genotypes using polymerase chain reaction (PCR) reaction according to Luise et al. (2019a, b). Briefly, genomic DNA was extracted from hair samples of sows and genotyped to identify polymorphic variants. Sows carrying the GG genotypes at FUT1 gene were considered for piglet enrolment. A further selection criterion was the absence of hemolytic *E. coli* in piglets feces. Microbiological analyses of selective mediums (Agar MacConkey) (Hayer et al. 2020; Li et al. 2020; Remfry et al. 2020) were thus carried out before transport and upon arrival on fecal samples collected from enrolled piglets.

### Animals and experimental design

Twenty-seven weaned piglets (28 ± 2 days) balanced per weight (9.79 ± 1.25 kg) and sex, were randomly allotted in four experimental groups in randomized complete block design and, after 7 days of adaptation period, fed ad libitum for the entire experimental period according to the following dietary treatments: control group (CTRL, n = 13) fed basal diet, phytogenic additive group 1 (PHY1, n = 7) fed basal diet supplemented with 200 g/100 kg phytogenic additive, phytogenic additive group 2 (PHY2, n = 7) fed basal diet supplemented with phytogenic additive plus 2000 ppm of short and medium chain fatty acids premix.

In order to achieve the same nutrient concentrations, the control group received basal diet supplemented with the same premix carrier used for treatment groups (95% wheat meal and 5% of coconut oil) without phytogenic compounds. The iso-energetic and iso-proteic diets (Table S1) were formulated (Plurimix; Fabermatica, CR, Italy) according to animal requirements for the post weaning phase defined by the US National Research Council (NRC 2012). The phytogenic feed additive (FRESTA®F, Delacon Biotechnik GmBH), approved by EU regulation (Reg. CE 1831/2003), as zootechnical additive, was composed of essential oil from caraway oil (d-carvone 3.5–6.0 mg/g) and lemon (limonene: 2.3—9.0 mg/g), dried herbs and spices (1.5% clove powder, 10% cinnamon powder, 1.5% nutmeg powder, 5% onion powder, 2% pimento powder, 5% orange peel powder, 12.5% peppermint powder and 12.5% chamomile powder). The SCFA and MCFA premix was composed by butyric (C4), caprylic (C8), capric (C10) and lauric acid (C12). The phytogenic products (with or without SCFA and MCFA) or the premix carrier were mixed with the compound diets for 30 min in order to ensure a homogeneous distribution. Diets have been provided in meal without any technological treatments, except for mixing procedure. During the mixing process the temperature was monitored in order to do not overcome 30 °C.

**Fig. 1:**
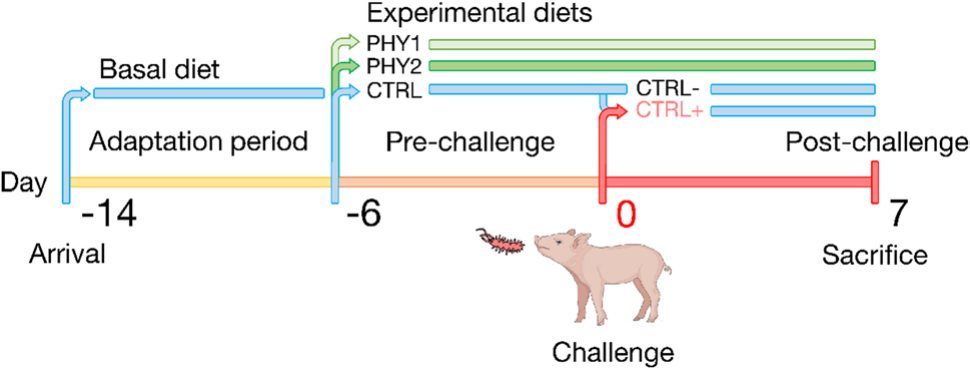
Experimental trial design from arrival (-14) to 7 days post-challenge. PHY1: treatment group fed basal diet supplemented with 200 g/100 kg of phytogenic additive; PHY2: treatment group fed basal diet supplemented with 200 g/100 kg of phytogenic additive supplemented with 2000 ppm of short and medium chain fatty acids premix; CTRL: group fed basal diet supplemented with premix carrier divided into negative control (CTRL-) and positive control (CTRL +) challenged at day 0.

Piglets were housed in two environmentally controlled rooms, in individual pens, with a plastic slatted floor and constant temperature (27° C) and humidity (60%) for the entire experimental period. The trial was divided into a pre- and post-challenge, considering the challenge as day 0 (Fig. 1).

### Chemical analysis of experimental diets

Diets were analyzed for proximate analysis, including moisture, crude protein (CP), crude fibre (CF), ether extract (EE), and ash. The moisture determination was performed by oven-drying at 65 °C for 24 h (Regulament EC 152/2009). Crude protein content was measured according to the Kjeldahl method (Association of Official Analytical Chemists method 2001.11). Crude fiber was determined by the filter bags technique (American Oil Chemistry Society 2009). Ether extract content was determined in a Soxhlet system after hydrolysis (Association of Official Analytical Chemists method 2003.05). Ash was measured using a muffle furnace at 550 °C (Association of Official Analytical Chemists method 942.05).

### Experimental challenge

*E. coli* challenger strain was genetically characterized by polymerase chain reaction (PCR) (Applied Biosystem 7500) in order to detect the presence of the two important virulence profile: subunit B of verocytotoxin type 2 and F18 adhesive fimbriae (Table 1).

**Table 1:** PCR conditions and oligonucleotide sequences of F18 adhesive fimbriae and VTe2 (B-subunit) encoding genes

Gene	Accession number (GenBank)	Size (pb)	Primer sequence (5’ to 3’)	PCR conditions
*F18 adhesive fimbriae*	AJ308332.1	519	5’GATCCATGAAAAGACTAGTGTTTATTTCTTTTG 3’CGAATGCGCCAATGAATGTTCATTCTCGAG	Den. 95 °C 1’ ann. 56 °C 1′20’’ ext. 72 °C 1′30’’ 35 cycles
*VTe2 (B-subunit)*	GU459254.1	270	5’GGATCCATGAAGAAGATGTTTATAGCGG 3’AACGGGTCCACTTCAAATGATTCTCGAG	Den. 95 °C 1’ ann. 50 °C 1′20’’ ext. 72 °C 1′30’’ 35 cycles

Twenty piglets, except for piglets in CTRL- group (n = 7), on day 0 (challenge day) were orally infected with O138 *Escherichia coli* F18 + strain obtained from a permanent collection of the University of Milan and previously characterized (Rossi et al. 2014b, 2021; Dell'Anno et al. 2020).

Sixty minutes before the challenge, the piglets were sedated with azaperone (2 mL/head, Stresnil®, Janssen Cilag Spa, Milan, Italy), thereafter 30 mL of a 10% bicarbonate solution was orally administered to neutralize gastric acid and to increase the survival rate of the challenger strain in the stomach. After 10–15 min, the inoculum was given orally in a single dose of 5 mL of bacterial medium with 2 × 10^9^ colony-forming unit (CFU) of challenger strain, using a 16G catheter (Rossi et al. 2021). Animals were fasted 3 h before and 3 h after the challenge. At the same time, piglets in CTRL- were orally inoculated with 5 mL of Luria Bertani (LB) medium to balance the level of stress associated with the oral challenge.

### Zootechnical performance, clinical and fecal score

Average daily feed intake (ADFI) was recorded daily from day -6 to day 7 by measuring the refusals. Body weight (BW) was recorded on day -6 (first day of experimental diets), day 0 (challenge day), day 4 and day 7 (sacrifice day). Average daily gain (ADG) and feed efficiency were also calculated.

Piglets were individually evaluated throughout the trial by clinical examination, including observation of behavioral disturbances. In particular, oedema, epiphora, respiratory and hair scores were evaluated through three-point scales (oedema score: 0 = normal, 1 = mild, 2 = severe; epiphora score: 0 = normal, 1 = mild, 2 = severe; respiratory score: 0 = normal, 1 = slightly quick, 2 = quick; hair/bristles score: 0 = smooth, 1 = lightly brushy, 2 = highly brushy) (Rossi et al. 2021). In addition, cyanosis, a blue or red discoloration of the skin, which may or may not be localized to small areas, was considered not as a specific skin condition but as a symptom of disease. From day -6 to day 7, all piglets were evaluated for the fecal score. Clinical signs of the disease were identified according to the point scale score described by Rossi et al. (Rossi et al. 2014b). A four-point scale was adopted to score fecal consistency: 0 = normal, 1 = soft consistency, 2 = mild diarrhea, 3 = severe diarrhea; considering > 1 as an indicative of diarrhea. Fecal color was evaluated using a three-point scale: 1 = yellow, 2 = green; 3 = brown.

### Microbiological evaluation of fecal samples

Individual fecal samples were collected from rectal ampulla from each piglet, on days -1, 1, 2, 3 and 4 to perform microbiological analysis and evaluate the challenger strain shedding. For each sample, 1 g of feces was homogenized with 1 ml of saline solution and incubated overnight at 37 °C on sheep blood agar plates 5% (Blood Agar Base No. 2-Oxoid) in order to examine the presence of hemolytic colonies. The total hemolytic bacteria count was performed by counting the number of colonies cultured from serial dilutions of each fecal sample in order to evaluate the presence of hemolytic *E. coli* in relation to the total bacteria population.

### Necropsy, intestinal samples, and histopathology

At day 7 post-challenge, sixteen animals (n = 4/treatment), were randomly selected and euthanized and tissue samples were collected for histopathological and molecular analyses of intestinal tissues.

Animal care and euthanasia procedures were conducted in accordance with the European Union guidelines (86/609/EEC) and approved by the Italian Ministry of Health. Briefly, selected piglets were sedated with 2 mL/head of azaperone (Stresnil®, Janssen Cilag SpA, Milan, Italy) intramuscularly. After 20 min, animals received a bolus injection of propofol intravenously in the right and left lateral auricular vein. Anesthesia was maintained with 40 mg/kg of tiletamine/zolazepam intramuscularly (Zoletil 100, Virbac UK, Bury Saint Edmund, England). Finally, unconscious animals were euthanized by the intracardiac administration of a solution with embutramide, mebezonium iodide and tetracaine hydrochloride (0.3 mg/kg, Tanax, MSD Animal Health, Boxmeer, Netherlands). The intestine of each animal was weighed, and intestinal samples of ileum at 1 cm from ileocecal valve, mesenteric lymph nodes were harvested. For the histological evaluation, samples were diluted in 10% neutral formalin buffer and stored at 4 °C. Tissues were rinsed with sterile saline solution and transferred into 2 mL cryotubes, snap-frozen in liquid nitrogen and stored at -80 °C until further analysis.

Histological examinations of collected intestinal and lymph nodes samples for each piglet were carried out. The fixed samples were embedded in paraffin, and 5 µm thick histological sections were performed with a microtome. Cross sections were stained with hematoxylin and eosin and were blind evaluated by light microscopy. A four-point scale was adopted for inflammatory infiltrates, epithelial regeneration, fusion of villi, oedema, hyperemia, necrosis of mucosa, T atrophy, stroma, and follicular hyperplasia; considering: 0 = no evidence; 1 = slight presence; 2 = moderate; 3 = severe. Samples of duodenum were collected and frozen in liquid nitrogen for gene expression analysis.

### Duodenum gene expression

Total RNA was extracted from the duodenum using FastGene Scriptase Basic (Nippon genetics) according to the manufacturer’s instructions. The integrity of total RNA was assessed by gel electrophoresis to detect the 18S and 28S rRNA bands. A combination of oligo-dT and random primers was used to reverse transcribe 100 ng of total duodenal RNA to cDNA (cDNA synthesis kit, FastGene Scriptase Basic, Nippon Genetics). Primer pairs were first tested for their specificity in qualitative PCR, using the pooled cDNA as a template. The cycling profile for the assay consisted of initial denaturation of RNA (65 °C × 5’), then the annealing of random primers (25 °C × 10’), followed by the annealing of oligo-dT and transcription (42 °C × 60’). At the end of the cycle, the enzyme deactivation (90 °C × 5’) was performed. The abundance of cytochrome c oxidase subunit I (COX1), cytochrome c oxidase subunit II (COX2), interleukin 10 (IL-10), interleukin 6 (IL-6), lysyl oxidase (LOX), glutathione peroxidase 2 (GPX2), NAD (P) H quinone dehydrogenase 1 (NQ01) claudin domain containing 1 (CLDND1) and occludin (OCLN) (Table 2) mRNA was determined using SYBR Green-based real-time quantitative PCR assays (7500 Fast Dx, Applied Biosystems). Only reaction efficiencies that were near to 100% were considered for further analysis. The mean values for the transcripts were normalized to the arithmetic mean of mRNA abundance of βactin as the reference gene within each sample. The comparative CT method was used to determine fold changes in gene expression, calculated as 2^−∆∆CT^. The final results were presented as the fold changes of target gene expression in a target sample relative to a reference sample, normalized to βactin rRNA (Livak and Schmittgen, 2001). The βactin rRNA was used to calculate the threshold cycles, since it previously showed constant values under all the conditions adopted.

**Table 2:** Primer sequences and relative amplicon dimensions

Gene^1^	Accession number (GenBank)	Size (pb)	Primer sequence (5’ to 3’)
βactin F	DQ845171	76 bp	CTACGTCGCCCTGGACTTC
βactin R	DQ845172		GCAGCTCGTAGCTCTTCTCC
IL-6 F	JQ839263	112 bp	TGGGTTCAATCAGGAGACCT
IL-6 R	JQ839264		CAGCCTCGACATTTCCCTTA
IL-10 F	L2001	105 bp	TGAAGAGTGCCTTTAGCAAGCTC
IL-10 R	L2002		CTCATCTTCATCGTCATGTAGGC
COX1 F	EF568726	102 bp	GGAGCGGGTACTGGATGAAC
COX1 R	EF568726		CACCTGCAAGGGTGTAGGGAGL
COX2 F	AF304201	141 bp	AAGACGCCACTTCACCCATC
COX2 R	AF304201		TCCATTGTGCTAGTGTGTGTCA
GPx2 F	DQ898282	103 bp	GGAGATCCTGAACAGCCTCA
GPx2 R	DQ898282		GCGAAGACAGGATGCTCATT
LOX F	NM_001164001	112 bp	GTGGAGCACGAAAGCAAGACCC
LOX R	NM_001164001		AAGGTGGGGTATGCATCGACAC
NQ01 F	NM_001159613	118 bp	ATCACAGGTAAACTGAAGGACCC
NQ01 R	NM_001159613		GCGGCTTCCACCTTCTTTTG
CLAUDIN1 F	NM_001244539	90 bp	TCTTTCTTATTTCAGGTCTGGCT
CLAUDIN1 R	NM_001244539		ACTGGGGTCATGGGGTCATA
OCCLUDIN F	NM_001163647	106 bp	GTCCACCTCCTTATAGGCCTGATG
OCCLUDIN R	NM_001163647		CGCTGGCTGAGAAAGCATTGG

^1^*CTB* actin beta, *IL-6* interleukin-6, *IL-10* interleukin 10, *COX1* cytochrome c oxidase subunit I, *COX2* cytochrome c oxidase subunit II, *LOX* lysyl oxidase, *GPX2* glutathione peroxidase 2, *NQ01* NAD (P) H quinone dehydrogenase 1, *CLDND1* claudin domain containing 1, *OCLN* occludin

### Blood Samples, serum metabolite profile and serum acute phase proteins

Blood was collected from the jugular vein of each animal on day -1, day 3 and day 7 through vacutainer tubes containing ethylenediaminetetraacetic acid (EDTA) and heparin as anticoagulants. Plasma was collected after centrifugation (3000 rpm, 10 min, 4 °C), aliquoted and stored at -20 °C for further analysis. Hematocrit was evaluated on whole blood using the microhematocrit method. The concentration of total protein (g/L), albumin (g/L), globulin (g/L), albumin/globulin (A/G ratio), alanine aminotransferase (ALT-GPT; IU/L), aspartate aminotransferase (AST-GOT; IU/L), phosphatase alkaline (ALP; IU/L), glucose (mmol/L), urea (mmol/L), total bilirubin (µmol/l), total cholesterol (mmol/L), calcium (mmol/L), phosphorus (mmol/L) and magnesium (mmol/L) were analyzed in serum via standard enzymatic colorimetric analysis through a multiparametric autoanalyzer for clinical chemistry (ILab 650; Instrumentation Laboratory Company, Lexington, MA, USA) at 37 °C by the Lombardy and Emilia Romagna Experimental Zootechnic Institute (IZSLER). Porcine C-reactive protein (CRP) concentration was determined in serum with a commercial sandwich immunoassay Kit (Mybiosource, San Diego, CA, USA) following the manufacturer’s instructions. The results were read at 450 nm using a microplate reader (Model 680, Bio-Rad Laboratories, CA, USA). Haptoglobin (HP) serum concentrations were measured through a colorimetric kit (PhaseTM Range porcine Haptoglobin Assay; Tridelta Development Ltd) according to the manufacturer’s instructions. The results were read at 630 nm on a microplate reader (Model 680, Bio-Rad Laboratories, CA, USA).

### Statistical analysis

Zootechnical performance and fecal microbiological analysis were analyzed using a linear model after testing the normality of data through Shapiro–Wilk test using JMP Pro 15® (SAS Inst. Inc., Cary, NC, USA). The model included the fixed effect of treatments (Trt), the effect of time (Time), and the interaction between treatment and time (Trt x Time).

Serum metabolites were evaluated performing analysis of covariance (ANCOVA) to adjust the initial variability of the pre-challenge period after testing the normality of data through Shapiro–Wilk test using JMP Pro 15® (SAS Inst. Inc., Cary, NC, USA).

Clinical score data were converted into a dichotomous variable (normal/pathological), and observed frequencies were assessed using the Chi-squared Test. Histological scores, intestinal weight and relative gene expression were analyzed using Kruskal–Wallis test (PROC NPAR1WAY of SAS 9.4 software) for non-parametric data due to the small sample size of euthanized animals at day 7. Multiple comparisons for parametric statistics were evaluated with the Tukey’s Honestly Significant Difference test (Tukey’s HSD) or Tukey–Kramer test and Steel–Dwass test was used for non-parametric multiple comparisons. The results were presented as least square means (LSMEANS) ± standard error (SE) for parametric data and as medians and range (minimum–maximum) for non-parametric results. Means or medians were considered statistically different when *p* ≤ 0.050 and statistical tendency was considered when *p* < 0.100.

## Results

### Chemical composition of the experimental diets

Proximate analysis of the experimental diets showed comparable contents of the principal nutrients. The inclusion of phytogenic based additives with or without MCFA and SCFA did not affect the nutrient balance of feed (Table S1).

### Zootechnical performance

During the pre-challenge period, no statistically significant differences among experimental groups were observed. Considering the entire post-challenge period, ADFI of CTRL + was lower than PHY2 and CTRL- (*p* < 0.005; Table 3).

**Table 3:** Zootechnical performance of experimental groups during the post-challenge period

	PHY1	PHY2	CTRL +	CTRL-	*p-value*		
	(n = 7)	(n = 7)	(n = 6)	(n = 7)	Trt	Time	Trt × Time
BW, kg							
d 0	10.39 ± 0.68	10.46 ± 0.68	10.23 ± 0.73	10.67 ± 0.68	0.342	< 0.001	0.963
d 4	11.29 ± 0.68	11.73 ± 0.68	11.25 ± 0.73	11.89 ± 0.68			
d 7	12.33 ± 0.68	12.96 ± 0.68	11.55 ± 0.73	13.42 ± 0.68			
ADG, kg/d							
d 1–4	0.22 ± 0.09	0.32 ± 0.09	0.26 ± 0.10	0.35 ± 0.09	0.083	0.323	0.262
d 5–7	0.35 ± 0.09	0.41 ± 0.09	0.10 ± 0.10	0.51 ± 0.09			
d 1–7	0.29 ± 0.06	0.36 ± 0.06	0.18 ± 0.07	0.41 ± 0.06			
ADFI, kg/d							
d 1–4	0.42 ± 0.04	0.46 ± 0.04	0.35 ± 0.05	0.44 ± 0.04	< 0.005	0.040	0.294
d 5–7	0.42 ± 0.04	0.56 ± 0.04	0.37 ± 0.05	0.58 ± 0.04			
d 1–7	0.42 ± 0.03^AB^	0.51 ± 0.03^B^	0.36 ± 0.03^A^	0.51 ± 0.03^B^			
FCR, kg/kg							
d 1–4	1.84 ± 0.41	1.59 ± 0.38	1.60 ± 0.41	1.41 ± 0.41	0.054	0.248	0.069
d 5–7	1.57 ± 0.04	1.40 ± 0.38	3.60 ± 0.58	1.32 ± 0.38			
d 1–7	1.70 ± 0.31	1.49 ± 0.27	2.60 ± 0.36	1.36 ± 0.28			

Data are presented as least squared means (LSMEANS) and standard errors (SE)

^A^^−^^B^ Different uppercase letters indicate statistically significant differences between treatment groups (*p* < 0.01)

*BW* body weight, *ADFI* average daily feed intake, *ADG* average daily gain, *FCR* feed conversion ratio, *Trt* treatment effect, *Time* time effect, *Trt* × *Time* interaction between treatment and time

### Influence of phytogenic treatments on clinical score, fecal consistency and color

During the pre-challenge period and at day 0, the piglets did not show significant differences among clinical scores, indicating a general good health status. Although statistical differences among treatments were not identified, several altered scores were registered from 1 to 4 days post-challenge. After experimental infection, considering the numerical differences of clinical score frequencies (considered as altered clinical conditions for a score of ≥ 1) revealed that the experimental procedures influenced the clinical status of piglets (Table 4). However, from 5 to 7 days post challenge, a non-normal hair score frequency tended to increase in CTRL + compared to the other experimental groups (9.52% for PHY1, 14.81% for PHY2, 33.33% for CTRL + and 14.29% for CTRL-; *p* = 0.071).

**Table 4:** Frequencies (expressed as percentages) of clinical score ≥ 1 from 1 to 7 days post-challenge

	Treatments				*p-value*
Days 1–4	PHY1 (n = 7)	PHY2 (n = 7)	CTRL + (n = 6)	CTRL- (n = 7)	
Hair	28.57	10.71	16.67	14.29	0.325
Respiratory	3.57	0.00	4.17	0.00	0.528
Oedema	3.57	3.70	8.33	0.00	0.471
Epiphora	3.57	14.29	8.33	7.14	0.536
Days 5–7	PHY1 (n = 7)	PHY2 (n = 7)	CTRL + (n = 6)	CTRL- (n = 7)	
Hair	9.52	14.81	33.33	14.29	0.071
Respiratory	0.00	0.00	0.00	0.00	-
Oedema	0.00	0.00	0.00	0.00	-
Epiphora	0.00	0.00	0.00	0.00	-

Data are presented as a percentage of clinical score ≥ 1 registered from day 1 to day 7 post-challenge

Fecal score and color were the most informative indicators during the post-challenge period (Table 5). Significant higher frequencies of altered fecal color were recorded in challenged groups compared to CTRL- from 1 to 4 days post-challenge (*p* < 0.050). Significant differences in the manifestations of diarrhea (fecal consistency ≥ 2) were observed from 5 to 7 days after the challenge. In particular, PHY1 had higher number of diarrhea cases compared to PHY2, CTRL + and CTRL-, and PHY2 had a lower incidence compared to CTRL + and PHY1 (*p* < 0.010).

**Table 5:** Frequencies (expressed as percentages) of fecal consistency ≥ 2 and fecal color = 1 registered 1 to 7 days post-challenge

	Treatments				
Days 1–4	PHY1 (n = 7)	PHY2 (n = 7)	CTRL + (n = 6)	CTRL- (n = 7)	*p-value*
Fecal consistency	42.86	28.57	25.00	21.43	0.319
Fecal color	57.14^A^	39.29^A^	37.50^A^	17.86^B^	0.027
Days 5–7	PHY1 (n = 7)	PHY2 (n = 7)	CTRL + (n = 6)	CTRL- (n = 7)	
Fecal consistency	80.95^A^	28.57^B^	61.11^C^	38.10^B^	0.003
Fecal color	90.48	71.43	94.44	71.43	0.114

Data are presented as a percentage of fecal consistency ≥ 2 and fecal color = 1 registered from day 1 to day 7 post-challenge

^A^^−^^B^^−^^C^Different uppercase letters indicate statistically significant differences among treatment groups (*p* < 0.01)

### Microbiological evaluation of feces and challenger strain shedding

**Fig. 2:**
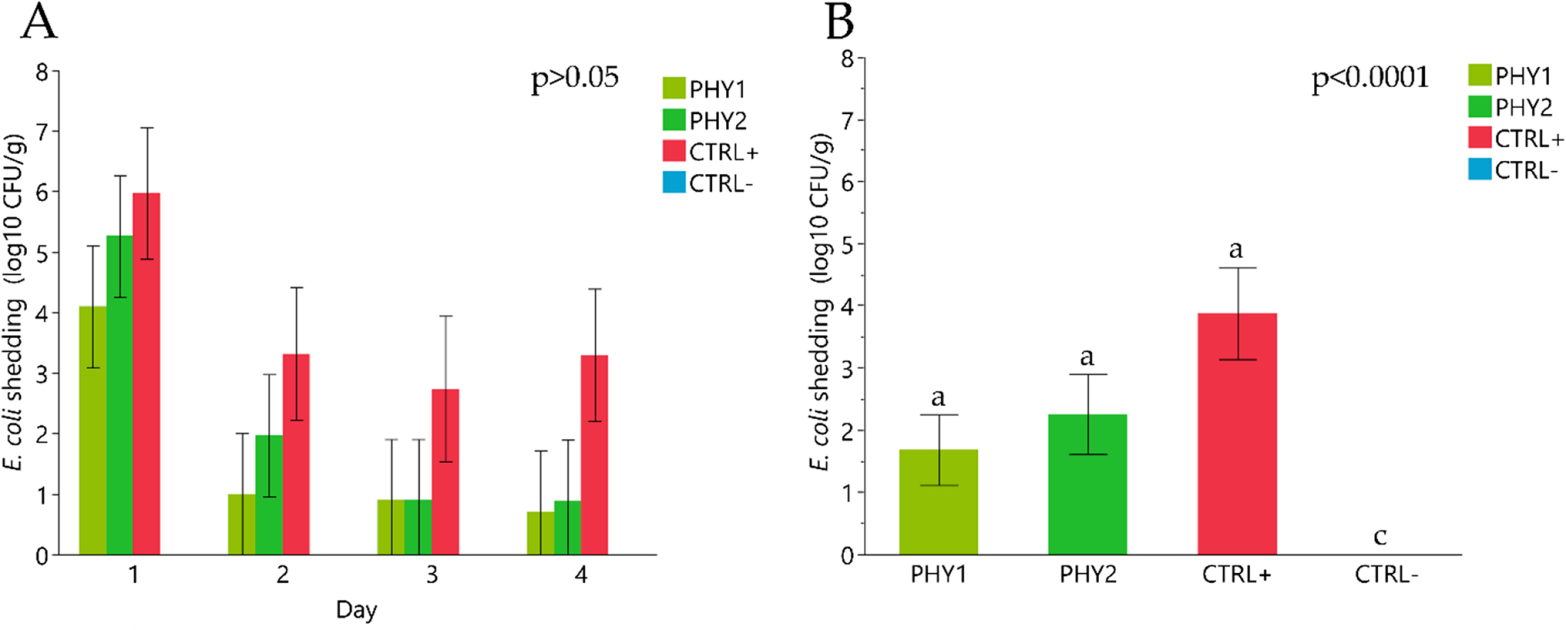
*Escherichia coli* fecal shedding during the four days post-challenge where **A**) presents daily hemolytic *E. coli* fecal shedding from day 1 to day 4 post-challenge; **B**) presents average fecal hemolytic *E. coli* fecal shedding from 1 to 4 days post-challenge. Data are presented as least squared means (LSMEANS) and standard errors (SE). ^A−B^ Different uppercase letters indicate statistically significant differences among treatment groups (*p* < 0.001)

Weaned piglets did not show the presence of challenger *E. coli* in feces during the adaptation period and on day 0. Total bacterial count did not show statistically significant differences among groups from 1 to 4 days after the challenge (8.37 ± 0.47 log_10_ CFU/g for PHY1, 8.04 ± 0.47 log_10_ CFU/g for PHY2, 7.73 ± 0.51 log_10_ CFU/g for CTRL + and 7.71 ± 0.47 log_10_ CFU/g for CTRL-). Also after the challenge, all the experimental groups (except for negative control, CTRL-) registered fecal shedding of challenger *E. coli* strain (Fig. 2). Statistically significant increased fecal shedding of hemolytic *E. coli* was observed in challenged groups compared to CTRL- from day 1 to day 4 post-challenge (4.09 ± 0.01 log_10_ CFU/g for PHY1, 5.25 ± 1.10 log_10_ CFU/g for PHY2, 5.95 ± 1.09 log_10_ CFU/g for CTRL + and 0.00 ± 1.01 log_10_ CFU/g for CTRL-; *p* < 0.001).

### Histological evaluation and gene expression

Samples were examined for the presence of inflammation both in villi and in lamina propria, epithelial regeneration, fusion of villi, oedema in deep lamina propria, T atrophy, stroma (fibroconnective and histiocytes), and follicular hyperplasia.

Intestinal weight results did not reveal significant differences between treatment groups after 7 days post-challenge (Table S2). Phytogenic dietary treatments did not significantly affect ileum inflammatory infiltrates, epithelial regeneration, oedema and hyperemia after 7 days (Table 6).

**Table 6:** Histological examination of ileum and lymphoid of weaned piglets fed experimental diets on day 7

Score	Treatments								*p-value*
	PHY1 (n = 4)		PHY2 (n = 4)		CTRL + (n = 3)		CTRL- (n = 4)		
	median	min–max	median	min–max	median	min–max	median	min–max	
Ileum									
Inflammatory infiltrates	1	0–2	2	2–3	2	1–2	1	1–2	0.068
Epithelial regeneration	0	0–0	0	0–0	0	0–0	0	0–0	1.000
Fusion of villi	1	0–3	3	2–3	2	1–3	1	0–2	0.223
Oedema	0	0–1	0	0–1	0	0–1	0	0–1	1.000
Hyperemia	1	0–1	1	1–2	0	0–2	1	0–1	0.382
Necrosis of mucosa	0	0–0	0	0–1	0	0–0	0	0–0	1.000
Lymphoid									
T atrophy	0	0–1	1	0–1	0	0–1	0	0–0	0.253
Stroma	0	0–1	1	1–2	2	0–3	0	0–1	0.073
Follicular hyperplasia	0	0–2	2	1–3	1	0–3	0	0–1	0.186

Data are presented as medians and minimum and maximum value (min–max)

Relative expressions of IL-10, IL-6, LOX, GPX2, NQ01 and CLDND1 were not affected by phytogenic dietary treatments (Table 7). The relative expression of occludin was downregulated at day 7 post-challenge (*p* < 0.012). Pairwise comparisons revealed only a tendency to increase in challenged groups compared to CTRL- (*p* = 0.066).

**Table 7:** Duodenum expression of the main genes related to the intestinal integrity, inflammation and health of weaned piglets fed experimental diets on day 7 post-challenge

Relative expression^a^	Treatment								*p-value*	
	PHY1 (n = 4)		PHY2 (n = 4)		CTRL + (n = 4)		CRTL- (n = 4)			
	median	min–max	median	min–max	median	min–max	median	min–max		
IL-6	0.32	0.22–0.43	0.48	0.08–0.87	0.34	0.23–1.10	1.00	1.00–1.00		0.139
IL-10	0.26	0.14–0.61	0.94	0.11–1.71	0.24	0.09–0.83	1.00	1.00–1.00		0.110
COX1	0.74	0.58–8.15	4.24	0.51–15.31	3.36	0.56–11.36	1.00	1.00–1.00		0.671
COX2	0.46	0.24–1.77	1.57	0.10–4.94	1.34	0.14–2.36	1.00	1.00–1.00		0.734
LOX	0.61	0.25–1.37	2.33	0.21–3.96	1.19	0.40–1.39	1.00	1.00–1.00		0.331
GPX2	1.04	0.38–1.93	2.98	0.31–3.53	2.11	0.25–6.33	1.00	1.00–1.00		0.426
NQ01	0.68	0.27–5.73	6.21	0.28–8.62	5.61	0.46–8.21	1.00	1.00–1.00		0.315
CLDND1	1.37	0.31–6.33	14.40	0.39–22.20	6.05	0.69–22.67	1.00	1.00–1.00		0.619
OCLN	0.42	0.02–0.96	0.34	0.09–0.72	0.55	0.14–0.96	1.00	1.00–1.00		0.012

Data are presented as medians and minimum and maximum value (min–max)

*IL-6* interleukin-6, *IL-10* interleukin 10, *COX1* cytochrome c oxidase subunit I, *COX2* cytochrome c oxidase subunit II, *LOX* lysyl oxidase, *GPX2* glutathione peroxidase 2, *NQ01* NAD (P) H quinone dehydrogenase 1, *CLDND1* claudin domain containing 1, *OCLN* occludin

^a^Relative expressions of selected genes are presented as 2-.^ΔΔCT^

### Influence of phytogenic treatments on hematological and serum metabolites

The serum metabolic profile did not show statistically significant differences between the experimental groups at day 3 after the challenge (Table S3). After 7 days post-challenge, a significantly higher level of total protein content was observed in CTRL + compared to CTRL- (*p* = 0.050) (Table 8). Globulin content tended to be higher in CTRL + than CTRL- at 7 days post-challenge (*p* = 0.055). PHY2 had a higher level of AST-GOT at day 7 compared to the other challenged groups (*p* < 0.050). Acute phase proteins were not affected by dietary treatments and experimental challenge and showed no statistically significant differences after 3- and 7-days post-challenge (Table S4).

**Table 8:** Serum metabolites of weaned piglets fed experimental diets on day 7 post-challenge

	Treatments				
Blood	PHY1 (n = 7)	PHY2 (n = 7)	CTRL + (n = 6)	CRTL- (n = 7)	*p-value*
Total protein, g/L	56.95 ± 2.67^AB^	54.04 ± 2.67^AB^	62.32 ± 2.89^A^	50.83 ± 2.67^B^	0.050
Hematocrit, %	26.03 ± 1.05	26.09 ± 1.05	25.00 ± 1.16	25.43 ± 1.05	0.880
Albumin, g/L	30.76 ± 2.85	32.05 ± 2.72	28.70 ± 3.06	26.11 ± 2.71	0.452
Globulin, g/L	28.31 ± 2.63	26.20 ± 2.61	35.31 ± 2.86	24.43 ± 2.61	0.055
A/G ratio	1.06 ± 0.09	1.09 ± 0.09	0.89 ± 0.09	1.14 ± 0.09	0.280
Urea, mmol/L	2.51 ± 0.30	2.09 ± 0.30	2.90 ± 0.33	1.75 ± 0.30	0.093
ALT-GPT, IU/L	26.51 ± 2.51	31.09 ± 2.54	24.52 ± 2.81	26.66 ± 2.54	0.375
AST-GOT, IU/L	39.11 ± 6.79^A^	72.02 ± 7.42^B^	37.82 ± 7.05^A^	43.59 ± 6.74^AB^	0.014
ALP, UI/L	170.30 ± 17.78	197.15 ± 17.71	149.83 ± 19.35	195.84 ± 18.32	0.262
Total bilirubin, µmol/l	2.25 ± 0.16	1.86 ± 0.16	1.86 ± 0.17	1.70 ± 0.16	0.123
Glucose, mmol/L	4.91 ± 0.28	5.52 ± 0.28	5.12 ± 0.31	4.91 ± 0.28	0.421
Total cholesterol, mmol/L	2.17 ± 0.11	2.21 ± 0.11	2.20 ± 0.12	2.19 ± 0.11	0.992
Calcium, mmol/L	2.55 ± 0.15	2.89 ± 0.15	2.49 ± 0.17	2.54 ± 0.15	0.243
Phosphorus, mmol/L	2.78 ± 0.11	3.00 ± 0.11	2.87 ± 0.12	3.04 ± 0.13	0.426
Magnesium, mmol/L	0.85 ± 0.04	0.91 ± 0.04	0.96 ± 0.04	0.87 ± 0.04	0.174

Data are presented as least squared means (LSMEANS) and standard errors (SE)

*A/G* albumin/globulin, *ALT-GPT* alanine aminotransferase, *AST-GOT* aspartate aminotransferase, *ALP* alkaline phosphatase, *HDL* high-density lipoprotein, *LDL* low density lipoprotein

^A^^−^^B^Different uppercase letters indicate statistically significant differences among treatment groups (*p* ≤ 0.05)

## Discussion

Weaning is a critical period where piglets need to adapt to a new diet, environment and to develop their own immunity (Tretola et al. 2019). During this phase, PWD is one of the major causes of gastrointestinal disorders leading to high morbidity, antibiotic use and economic losses. Several natural extracts have been investigated for their functional proprieties to decrease diarrhea occurrence in piglets, with discordant results. The general aim of this study was to evaluate the protective effect of innovative phytogenic premix with or without MCFA and SCFA against O138 *E. coli* in weaned piglets. Genetic characterization of the sows led to the enrollment of piglets that were potentially susceptible to F18 fimbriae. In fact, the presence of F18 receptor (F18R) on porcine intestinal epithelium is crucial for the development of *E. coli* infections.

During the pre-challenge period (day -6 to day 0), the piglets showed comparable growth performance, demonstrating that the supplementation of additives did not influence their growth and feed consumption or feed palatability. Even if the effect on zootechnical performance was limited by the short experimental period (EFSA 2018), ADFI was affected by the treatment.

However, ADFI of CTRL + was significantly lower compared to PHY2 and CTRL- groups. The observed decrease in feed intake of CTRL + suggests that the challenged group without any supplementation reduced the feed consumption probably due to the detrimental effect of experimental infection. In addition, higher dietary intake is often related to a better health status (Czech et al. 2021), indicating that the treatment with PHY and organic acids could have supported animals’ health resulting in increased feed intake during the entire post-challenge period. PHY2 group showed a similar performance to CTRL- (uninfected), suggesting that dietary supplementation with the phytogenic premix, MCFA and SCFA was very effective in dealing with O138 *E. coli* infection, thus supporting intestinal health of animals. The addition of MCFA and SCFA may enhance animal growth by several mechanisms as previous studies described (e.g. inhibitory activity, mucosal epithelium integrity support) (Royce et al. 2013; Ferrara et al. 2017; Diao et al. 2019). In addition, phytogenic feed additives derived from spices and herbs are commonly used in animal nutrition as an alternative to in-feed antibiotics due to their antibacterial, antiviral and antioxidant properties. These effects are generally due to the presence of different bioactive compounds such as alkaloids, flavonoids, glycosides, mucilage, saponins, tannins, phenolics, polyphenols, terpenoids, and polypeptides (Upadhaya et al. 2016; Nowak et al. 2017; Caprarulo et al. 2020a; Dell’Anno et al. 2020; Reggi et al. 2020). Our results are in line with other studies demonstrating the antibacterial activity of PHYs, MCFA and SCFA on a wide range of pathogens (Dibner and Buttin 2002; Salsali et al. 2008).

In terms of clinical examination, from day 1, clinical scores were affected by experimental infection, confirming that disease development impaired the clinical status of challenged animals compared to the pre-challenge period. Moreover, significant differences in pathological hair, respiratory, oedema and epiphora scores were not detected in infected groups. This was probably due to the individual variability and the small sample size that could prevented to observe differences among groups. The O138 *Escherichia coli* challenger strain can impair gut health due to its capacity to adhere to the intestinal epithelium by specific fimbriae which could be followed by verocytotoxin production (Rossi et al. 2012, 2013) and in consequence may show systemic symptoms.

A slightly different situation was found during the evaluation of the fecal score and incidence of diarrhea. Experimental challenge affected transitory the fecal color and consistency during the 7 days post-challenge. Firstly, from day 1 to day 4 post-challenge, feces of yellowish color were registered more frequently in challenged group compared to CTRL- typically related to gastrointestinal disorders (Rossi et al. 2012). Considering total diarrhea cases recorded among experimental groups, from day 5 to day 7 the highest diarrhea frequency was registered, suggesting a late effect of challenge on fecal consistency compared to fecal color. These data are confirmed by a previous study by Rossi et al. (Rossi et al. 2021) showing that O138 *E. coli* experimental infection increased the sum of fecal score from 3 to 9 days post-challenge. Particularly, the highest diarrhea occurrence was observed in PHY1 compared to other groups, while PHY2 showed a fecal consistency comparable to CTRL- suggesting the counteracting activity of the phytogenic additives, SCFA and MCFA against experimental infection. Even if antibacterial activity of phytogenic additives was reported (Namkung et al. 2004), the observed effect on diarrhea incidence was probably related to their combined effect with SCFA and MCFA. It has been demonstrated, that SCFA and MCFA can exert an inhibitory activity (Lei et al. 2017; Swanson et al. 2018; Zhang et al. 2020) or enhance the functional properties of phytogenic additives (McKnight et al. 2019).

In addition, dietary supplementation of organic acids can modulate the intestinal environment, creating undesirable environmental conditions for pathogenic bacteria, thus also influencing the intestinal microbiota (Verstegen and Williams 2002). Even if is difficult to establish the exact mechanisms for the enhancing antimicrobial effect by the combination of PHYs with organic acids (SCFA and MCFA) in pigs, we can suppose that PHYs can act as a permeabilizing complex and modify pores of the bacterial wall, thus facilitating the entrance of organic acids with antimicrobial activity (Tugnoli et al. 2020). In addition, the reduction in undigested feed protein by organic acids reduces the negative fermentative processes, increases growth performance and repairment of damaged intestinal tissues (Jia et al. 2020). Our results suggest that the addition of MCFA and SCFA to the phytogenic premix significantly inhibited enterotoxigenic *E. coli* diarrhea, thus supporting intestinal health of animals.

Considering the challenger strain shedding, the proliferation started gradually from the day of challenge in line with clinical observations. Compared with the uninfected control group infected animals showed hemolytic *E. coli* shedding from day 1 post-challenge, thus confirming the success of the experimental infection.

Histopathological examination of the ileum, jejunum and large intestine is thus used to highlight clinical signs of *E. coli* infection (Luppi 2017). In our study, histological evaluation of the ileum and lymphoid of intestinal tissues did not reveal significant lesions. The animals in the experimental trial thus did not show severe signs of intestinal lesions. Although more frequent lesions were registered in the PHY2 group, these did not impair animal performance and there was a comparable growth curve to CTRL-. This was probably due to the supplementation of phytogenic with SCFA and MCFA which could have supported intestinal health.

Gene expressions showed a high individual variability in terms of inflammatory parameters and tight junctions (TJs), probably due to the limited number of animals. We thus analyzed the expression of the TJ transmembrane protein (occludin) and the observed data were in line with morphological analyses. Our findings suggested that tight junction integrity tended to be disrupted seven days after infection in challenged groups compared to the CTRL-. Intestinal permeability is regulated by the tight junctions which are a primary determinant of epithelial paracellular permeability (Zhang et al. 2021). Disruption of occludin regulation is related to many diseases. During the inflammation process, specific domains of occludin are in fact thought to mediate the transepithelial migration of neutrophils across the TJ (Feldman et al. 2005). Inflammation produces effects on epithelial barriers, increasing the leakiness of occludin, and decreasing the barrier function of this protein. Occludin responds earlier to oxidative stress than claudin, which responds later to reactive oxygen species (ROS) (Blasig et al. 2011). Intestinal bacterial infection is associated with intestinal epithelial and crypt architectural irregularity and with barrier dysfunction, leading to an increase in intestinal mucosal permeability. The observed slight downregulation of occludin after seven days in challenged groups could be due to the harmful activity of the challenger strain. Further investigations are required to better understand the effect of PHYs, SCFA and MCFA on the modulation of genes involved in inflammation and intestinal integrity.

Considering the biochemical parameters of the experimental groups (PHY1, PHY2, CTRL + and CTRL-), the values were within the reference range of weaned pigs (Klem et al. 2010; IZSLER 2017), thus confirming that phytogenic additive supplementation had no detrimental effect on serum metabolism. The metabolite profile showed an increased level of total protein and a higher globulin content in CTRL + compared to CTRL-. However, globulin together with albumin are the two major constituents of serum proteins, which play a crucial role in the inflammatory process (Balan et al. 2020; Wang et al. 2020). The increase in globulin could be associated with an inflammatory process probably due to the experimental *E. coli* infection leading to an increased concentration of total serum proteins. The serum AST-GOT level is a specific marker for liver tissue and represents a valuable indicator for acute hepatocyte injury or cell membranes damage (Kim 2020; Amirabagya et al. 2021). Although our results are in line with the proper range of pig physiology parameters (Klem et al. 2010; IZSLER 2017; Caprarulo et al. 2020b), AST-GOT was probably higher in the PHY2 group due to the presence of SCFA and MCFA which are immediately available for hepatic metabolism. In fact, short-chain fatty acids can activate lipid and glucose metabolism independently of the pig gut microbiota (Zhou et al. 2021).

## Conclusions

Our study showed that phytogenic additive dietary supplementation limited the detrimental effect of experimental challenge. Phytogenic premix plus SCFA and MCFA revealed a positive effect on animal performance and health improving ADFI and fecal consistency during the post-challenge period compared to infected control group, suggesting that the combination of PHYs and organic acids can be considered as effective against pathogenic *E. coli* strains of weaned piglets. Due to the lack of studies on the argument, at this stage is too early to state that phytogenics are effective. Future studies will be necessary to confirm our results and extensively investigate how phytogenic additives and organic acids affect gene expression over time.

## Supplementary Information

Below is the link to the electronic supplementary material.

Supplementary file1 (DOCX 28 kb)

## Data Availability

All data generated or analyzed in this study are available from the corresponding author upon reasonable request. Data are stored on Google Drive at the following link: https://drive.google.com/drive/folders/1fpkkuaELR6a5ZIeTh3gszVoMrQJcGcMF?usp=sharing.
