# Supplementary material for: Protective effect of phytogenic plus short and medium-chain fatty acids-based additives in enterotoxigenic Escherichia coli challenged piglets
Source: Vet Res Commun. 2022 May 26;47(1):217–31. doi: 10.1007/s11259-022-09945-0 (PMC9873745; doi:10.1007/s11259-022-09945-0)
Supplement: Supplementary file 1 — Supplementary file1 (DOCX 28 kb) [file 11259_2022_9945_MOESM1_ESM.docx]

**Supplementary Materials**

**Table S1.** Ingredient composition of the experimental diets divided per control (CTRL) and treatment groups (TRT).

| **Ingredients, as % of fed basis** | **CTRL** | **TRT** |
| --- | --- | --- |
| Barley meal | 22.80 | 22.80 |
| Wheat meal | 12.33 | 12.33 |
| Milk whey powder | 11.00 | 11.00 |
| Corn meal | 6.50 | 6.50 |
| Corn flakes | 6.50 | 6.50 |
| Wheat flakes | 6.00 | 6.00 |
| Soybean protein | 5.50 | 5.50 |
| Dextrose monohydrate | 5.00 | 5.00 |
| Barley flakes | 4.00 | 4.00 |
| Plasma meal | 3.50 | 3.50 |
| Soybean meal | 3.50 | 3.50 |
| Wheat bran | 3.00 | 3.00 |
| Fish meal | 2.50 | 2.50 |
| Coconut oil | 1.50 | 1.50 |
| Soy oil | 1.50 | 1.50 |
| Fiber | 1.00 | 1.00 |
| Acidifiers | 0.80 | 0.80 |
| L-Lysine | 0.55 | 0.55 |
| Dicalcium phosphate | 0.50 | 0.50 |
| Benzoic acid | 0.40 | 0.40 |
| Calcium carbonate | 0.40 | 0.40 |
| L-threonine | 0.25 | 0.25 |
| Vitamins^1^ | 0.25 | 0.25 |
| DL-methionine | 0.23 | 0.23 |
| Sodium butyrate | 0.20 | 0.20 |
| Sodium chloride | 0.15 | 0.15 |
| L- Tryptophan | 0.08 | 0.08 |
| Copper sulphate | 0.06 | 0.06 |
| **Phytogenic premix^2^** | - | 0.20 |
| **Carrier premix^3^** | 0.20 | - |
| **Calculated nutrient composition^4^** |  |  |
| Crude protein (%) | 18.06 | 18.06 |
| Fat (%) | 4.92 | 4.92 |
| Crude fiber (%) | 2.88 | 2.88 |
| Ashes (%) | 6.04 | 6.04 |
| DE (Mc/Kg) | 3.47 | 3.47 |

^1^Additives per kg of diet: 10.000 UI vitamin A, 1.000 UI vitamin D3, 100 mg UI vitamin E, 3 mg vitamin B1, 96.3 mg vitamin B2, 5.8 mg vitamin B6, 27 mg vitamin B5, 0.040 mg vitamin B12, 4.8 mg vitamin K3, 0.19 mg biotin, 35 mg niacinamide, 1.4 mg folic acid, 120 mg choline chloride, 70 mg betaine chloride, 108 mg Fe as FeCO_3_, 38.5 mg Mn as MnO_2_, 112 mg Zn as ZnO, 19.3 Cu as CuSO_4_, 0.58 I as Ca(IO_3_)_2_, 0.29 Se as Na_2_SeO_3_.

^2^Phytogenic premix (FRESTA®F, Delacon Biotechnik GmBH) divided per PHY1 and PHY2 characterized by caraway oil ≥ 0.75% (d-carvone: 3.5-6.0 mg/g), lemon oil ≥ 0.75% (limonene: 2.3 - 9.0 mg/g), clove powder: 1.5%, cinnamon powder: 10%, nutmeg powder: 1.5%, onion powder: 5%, pimento powder: 2%, orange peel powder: 5%, peppermint powder: 12.5%, chamomile powder: 12.5% differentiable only for the inclusion of 2000 ppm of short and medium chain fatty acids premix in PHY2 additive.

^3^Carrier premix was supplemented to control group in order to achieve the same nutrient concentrations. Carrier composition: 95% wheat meal and 5% of coconut oil.

^4^Nutrient and digestible energy were calculated through Plurimix software (Fabermatica, CR, Italy) and expressed as fed basis content.

Analyzed chemical composition (% as fed): PHY1 = 19.12% crude protein, 4.06% ether extract, 2.35% crude fiber, 5.18% ash; PHY2 = 18.85% crude protein, 3.63% ether extract, 2.35% crude fiber, 5.31% ash; CTRL+ = 19.03% crude protein, 4.08% ether extract, 2.42% crude fiber, 5.13% ash; CTRL- = 18.47% crude protein, 4.04% ether extract, 2.30% crude fiber, 5.07 ash.

**Table S2.** Intestinal weights of weaned piglets fed experimental diets on day 7.

|  | **Treatment** | | | | | | | |  |
| --- | --- | --- | --- | --- | --- | --- | --- | --- | --- |
|  | **PHY1 (n=4)** | | **PHY2 (n=4)** | | **CTRL+ (n=4)** | | **CTRL- (n=4)** | | ***p- value*** |
|  | median | min-max | median | min-max | median | min-max | median | min-max |  |
| **Intestinal**  **weight** | 1.14 | 0.99-1.68 | 2.22 | 1.18-1.56 | 1.19 | 1.04-1.32 | 1.42 | 1.35-1.43 | 0.157 |

Data are presented as medians and minimum and maximum value (min-max).

PHY1: basal diet plus 200g/100kg of phytogenic additive (FRESTA®F, Delacon Biotechnik GmBH); PHY2: basal diet plus 200g/100kg of phytogenic additive (FRESTA®F, Delacon Biotechnik GmBH) plus 2000 ppm of short and medium chain fatty acids premix; CTRL: basal diet plus premix carrier divided per negative control (CTRL-) and positive control (CTRL+) challenged at day 0.

**Table S3**. Serum metabolites of weaned piglets fed experimental diets on day 3 post-challenge.

|  | **Treatments** | | | |  |
| --- | --- | --- | --- | --- | --- |
| **Blood** | **PHY1**  **(n=7)** | **PHY2**  **(n=7)** | **CTRL+**  **(n=6)** | **CRTL-**  **(n=7)** | ***p-value*** |
| Total protein, g/L | 55.56±2.31 | 53.11±2.31 | 54.52±2.49 | 53.31±2.30 | 0.852 |
| Hematocrit, % | 29.17±1.58 | 24.40±1.58 | 25.44±1.74 | 25.73±1.58 | 0.195 |
| Albumin, g/L | 28.72±1.16 | 28.03±1.11 | 26.76±1.25 | 27.86±1.11 | 0.744 |
| Globulin, g/L | 26.17±1.90 | 25.31±1.89 | 28.55±2.07 | 25.31±1.89 | 0.366 |
| A/G ratio | 1.15±0.09 | 1.12±0.08 | 1.02±0.09 | 1.16±0.08 | 0.698 |
| Urea, mmol/L | 2.26±0.36 | 2.76±0.36 | 2.65±0.39 | 2.35±0.36 | 0.733 |
| ALT-GPT, IU/L | 24.82±2.09 | 28.42±2.11 | 24.23±2.34 | 25.14±2.11 | 0.533 |
| AST-GOT, IU/L | 55.68±12.35 | 60.14±13.50 | 46.87±12.82 | 50.58±12.25 | 0.894 |
| ALP, UI/L | 117.96±18.03 | 193.94±17.95 | 151.60±19.62 | 183.88±18.58 | 0.460 |
| Total bilirubin, µmol/l | 2.14±0.17 | 2.24±0.18 | 1.99±0.19 | 2.08±0.17 | 0.801 |
| Glucose, mmol/L | 5.76±0.41 | 5.52±0.41 | 5.25±0.44 | 5.06±0.41 | 0.666 |
| Total cholesterol, mmol/L | 2.25±0.16 | 2.15±0.16 | 1.85±0.18 | 2.18±0.16 | 0.406 |
| Calcium, mmol/L | 2.58±0.04 | 2.55±0.04 | 2.46±0.05 | 2.54±0.04 | 0.299 |
| Phosphorus, mmol/L | 2.73±0.10 | 3.01±0.09 | 2.88±0.11 | 2.79±0.11 | 0.118 |
| Magnesium, mmol/L | 0.79±0.03 | 0.87±0.03 | 0.86±0.03 | 0.91±0.03 | 0.118 |

Data are presented as least squared means (LSMEANS) and standard errors (SE).

A/G = albumin/globulin; ALT-GPT = alanine aminotransferase; AST-GOT = aspartate aminotransferase; ALP = alkaline phosphatase; HDL = high-density lipoprotein; LDL = low density lipoprotein.

PHY1: basal diet plus 200g/100kg of phytogenic additive (FRESTA®F, Delacon Biotechnik GmBH); PHY2: basal diet plus 200g/100kg of phytogenic additive (FRESTA®F, Delacon Biotechnik GmBH) plus 2000 ppm of short and medium chain fatty acids premix; CTRL: basal diet plus premix carrier divided per negative control (CTRL-) and positive control (CTRL+) challenged at day 0.

**Table S4**. Serum acute phase protein (APPs) concentrations of weaned piglets fed experimental diets on day 3 and day 7 post-challenge.

|  |  | **Treatments** | | | |  |
| --- | --- | --- | --- | --- | --- | --- |
|  | **Acute phase protein** | **PHY1**  **(n=7)** | **PHY2**  **(n=6)** | **CTRL+**  **(n=6)** | **CTRL-**  **(n=7)** | ***p-value*** |
| Day 3 | HP, mg/ml | 2.94±0.57 | 1.91±0.64 | 2.30±0.61 | 2.40±0.57 | 0.679 |
|  | Hp/Total protein, % | 5.45±1.20 | 3.83±1.34 | 3.94±1.27 | 4.62±1.19 | 0.774 |
|  | CRP, ug/ml | 3.27±1.12 | 3.79±1.01 | 4.23±1.47 | 6.82±1.01 | 0.127 |
|  | CRP/Total protein, % | 6.14±2.46 | 7.41±2.21 | 7.54±3.19 | 12.71±2.21 | 0.234 |
| Day 7 | HP, mg/ml | 2.23±0.49 | 2.65±0.54 | 2.36±0.53 | 2.76±0.46 | 0.857 |
|  | Hp/Total protein, % | 4.15±1.04 | 4.95±1.15 | 3.88±1.11 | 5.51±0.96 | 0.681 |
|  | CRP, ug/ml | 4.57±0.99 | 5.03±0.75 | 1.47±1.14 | 3.61±1.01 | 0.111 |
|  | CRP/Total protein, % | 7.73±1.79 | 9.35±1.34 | 2.15±2.05 | 6.84±1.81 | 0.075 |

Data are presented as least squared means (LSMEANS) and standard errors (SE).

Hp: haptoglobin; Hp/total protein: haptoglobin/Total protein; CRP: Porcine C-reactive protein; CRP/Total protein: Porcine C-reactive protein/Total protein.

PHY1: basal diet plus 200g/100kg of phytogenic additive (FRESTA®F, Delacon Biotechnik GmBH); PHY2: basal diet plus 200g/100kg of phytogenic additive (FRESTA®F, Delacon Biotechnik GmBH) plus 2000 ppm of short and medium chain fatty acids premix; CTRL: basal diet plus premix carrier divided per negative control (CTRL-) and positive control (CTRL+) challenged at day 0.
